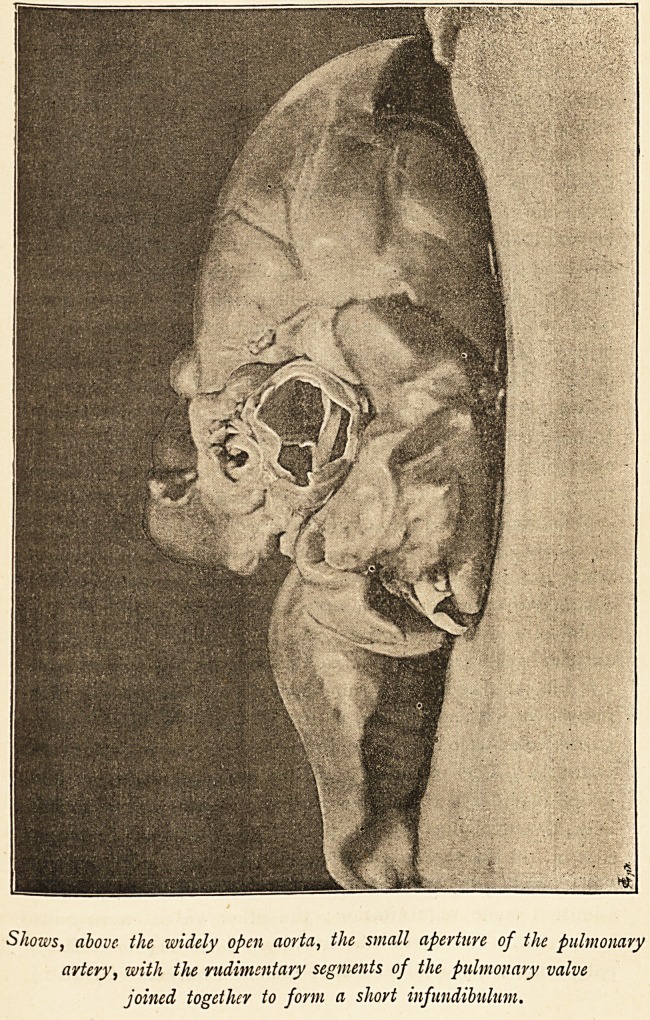# A Case of Myxœdema. Congenital Heart Disease. Disseminated Sclerosis

**Published:** 1889-12

**Authors:** J. Michell Clarke

**Affiliations:** Assist. Physician to the Bristol General Hospital, and Assist. Lecturer on Physiology, Bristol Med. School


### Clinical Records.

---

### A CASE OF MYXŒDEMA. CONGENITAL HEART DISEASE. DISSEMINATED SCLEROSIS.

By J. Michell Clarke, M.A., M.B., M.R.C.P., Assist. Physician to the Bristol General Hospital, and Assist. Lecturer on Physiology, Bristol Med. School.

**A Case of Myxœdema.**—Anne W., æt. 51, married, belongs to the working class, and lives in a poor low-lying district of Bristol. She states that her grand-parents were healthy and lived to old age, but she can only remember one of them. Her father died of an ulcered leg, æt. 79; her mother of apoplexy, at 58. She has six brothers and sisters, all healthy; two died in infancy. She has had nine children, four of whom are alive and well: one was stillborn, and the other four died at birth, or soon afterwards; the latter were probably born prematurely, but no positive evidence of this could be obtained. Apart from this, there was no history of syphilis.

She always suffered from attacks of megrim, coming on about once a month; but of late years has been very much better in this respect. She suffered very much at the birth of her first child, when there was severe hæmorrhage, and states that she has never been so strong since. She has had no other illness. The climacteric appeared at the age of 37. Her present illness, according to her own and her daughterin-law's account, dates from the climacteric period. Since then she has complained of weakness, nervousness, giddiness, drowsiness by day, with want of sleep at night, and a constant feeling of cold. These symptoms have gradually grown worse up to the present time, more especially the weakness. She is not distinctly worse in cold weather.

The patient is a small woman, well nourished, but not fat. An absence of expression about her features gives her a dull, heavy look. The face is pallid, except for a patch of slight redness over the cheek-bones; both the lower and the upper eyelids are œdematous, but do not pit on pressure, and have a peculiar transparent, waxy appearance. The nose is broad and clumsy, the mouth wide, and the eyebrows somewhat raised. The hair is thin, dry, and brittle; the teeth carious. There is a puffiness about the ankles, but no marked appearance of œdema either here or at the wrists. The skin is dry, and over the hands and feet thick; on the backs of the hands it is bright red from capillary congestion, covered with numerous cracks, and there is excessive desquamation of the cuticle both here and on the dorsum of the feet. The hands and fingers are small, and not spade-shaped. The soft palate appears to be rather large, and the tongue too big for the mouth.

No thyroid could be felt; and as she is not a fat woman, this region could be better explored than in most cases of myxœdema. The supra-clavicular regions are a little prominent, but contain no masses of fat. The chest is small and the abdomen rather large; the thoracic and abdominal organs are normal. The temperature taken on several occasions was 97.5°. The pulse is very weak, small, and of low tension. She suffers from constipation, and from flatulent dyspepsia. The urine is pale, acid, specific gravity 1014—1016, and contains no albumen. The muscles are normal; the deep reflexes sluggish; the superficial normal. Sensation is perfect, but distinctly delayed. She is slow in understanding what is said to her, and in answering questions; but otherwise her intelligence is good. Her mental state is generally placid; but she is irritable at times, and is very suspicious. For instance, her daughter-in-law came to the Hospital with her one day, and made the patient exceedingly angry by volunteering some statements about her; she would not leave her daughter alone with me, on account of what she might say about her. She has a fixed hallucination that there is a man under her bed; generally she can overcome this, but on several occasions has called her neighbours to turn him out. She sleeps lightly, and is much troubled by startling dreams. She also complains of agoraphobia.

Speech is peculiar, quite clear and distinct, but slow, drawling, deliberate, and very monotonous; rather nasal and syllabic. All her movements are slow and deliberate: she walks slowly and waddles from side to side as she goes; this is the more noticeable as she is a small woman. She stated that she had rather a narrow throat in swallowing, but complained of no other difficulty in deglutition. As to the special senses, smell, taste, and sight are natural. She is slightly deaf, and complains of noises in the ears. Mr. Pickering, the aural surgeon to the hospital, who kindly examined her ears for me, states that the deafness is of nervous origin. The pupils act well to light and to accommodation, and there is no paralysis of any kind. Her memory is impaired.

Her daughter-in-law subsequently told me that her relations have noticed gradual changes in her since she passed the climacteric, and that these changes have progressed more rapidly during the last six years. Her appearance has altered so much, that anyone who had formerly known her and had not seen her for the last ten years would now hardly recognise her. She has also lost flesh. Her relations have noticed the alterations in speech and gait during the last six years; her manner has changed, becoming apathetic, and she talks more childishly. Before this she spoke like other people, and was brisk and active in her habits. Now she complains much of cold, always wears a thick shawl in the house, and sits close to the fire, to which is attributed the redness of the backs of the hands. If she is not sitting over the fire, she wanders aimlessly about the house, and has a constant delusion that there is a man in her bedroom. She is also very suspicious as to her relations' intentions with regard to her. As to treatment, she has been taking iodide and bromide of potassium in an iron mixture, and has improved to some extent.

In conclusion, the appearance of the woman's face, her speech, gait, and mental condition present in marked degree the characteristic conditions proper to myxœdema. The extremities have so far escaped, not showing the morbid changes so commonly found in this disease.

---

**Congenital Heart Disease.**—The patient, a girl of 3, was brought to the hospital suffering from an extreme degree of cyanosis; she had been taken ill only a few hours previously with convulsions. Her mother stated that she had always been delicate and of a "bad colour." At the

*Shows the left ventricle laid open, and in upper part of septum ventriculorum a large opening divided into two parts by a muscular pillar, the upper opening lying just below the aortic valve.*

*Shows, above the widely open aorta, the small aperture of the pulmonary artery, with the rudimentary segments of the pulmonary valve joined together to form a short infundibulum.*

autopsy the body was badly nourished; there were a large number of small, pale, somewhat hard papules on the skin of the loins and back, and on the outer side of the lower extremities. There were patches of eczema on the head, and an old scar, $\frac{1}{2}$ inch in length, on the forehead. Pleural and pericardial sacs were healthy, containing a little serous fluid. The lungs weighed $3\frac{3}{4}$ ozs. each: they were small, pale, and very bloodless; the bronchial mucous membrane was normal. The heart was relatively large (to the size of the body). The right auricle and ventricle larger than the left, and had undergone much dilatation and hypertrophy, most marked in the case of the ventricle. The tricuspid orifice admitted index and middle fingers; mitral, only middle finger. The left auricle and ventricle small. The aorta and valves normal; the pulmonary valve was very small—about $\frac{1}{5}$ the aortic in diameter, or about $\frac{1}{4}$ inch. The segments only partially closed the opening, and were minute. There was an opening, $\frac{1}{2}$ by $\frac{1}{4}$ inch across, occupying upper part of septum, and divided into two by a muscular pillar descending vertically across it. In the unopened heart, a current of water passed into the right auricle escaped through the aorta, with the exception of a small stream through the pulmonary artery; similarly, water passing from the left auricle, besides the main stream issuing from the aorta, sent a feeble jet through the pulmonary artery. A good deal of *post mortem* clot was found in the right side, and some *ante mortem* clot also; only a little *post mortem* clot in left ventricle. The pulmonary valves were rudimentary, and joined together at their edges, forming a short infundibulum, with a central opening about $\frac{1}{12}$ inch wide. On the right side of the valve were two small granulations, the size of millet seeds, projecting into the opening. Pulmonary valve admitted some regurgitation; the other valves were quite competent. The aorta was healthy. *Liver* $11\frac{3}{4}$ ozs., fatty, pale, tough; gall-bladder full of bile, and normal. *Spleen* $1\frac{1}{2}$

ozs., dotted over on cut surfaces with little white rounded bodies resembling sago; spleen otherwise tough and congested. *Brain* weighed 26 ozs.: was very pale, but healthy. The kidneys, stomach, and intestines were healthy.

---

**Disseminated Sclerosis.**—J. B., a shipwright of 45 years of age, complained of tremor of the hands: very much worse when at work. The illness began with an attack of congestion of the lung 14 weeks ago, when on a voyage home from the East Indies; before this his hands had always been perfectly steady. He was a strongly-built man, but looked considerably older than his stated age. He also complained of pains on each side of the chest and head, and of a cough, and had lost 28 lbs. in weight during the last three months. After resting for some time, and at night, the tremor of the hands completely disappeared; but during short intervals of rest, and during the time I saw him, there was a more or less continuous very fine tremor, which when he attempted to do anything passed into a series of strong clonic spasms, increasing in intensity. He upset nearly the whole of a tall glass of water in carrying it to his mouth, and only succeeded in taking a mouthful. He had equal difficulty when asked to touch the tip of his nose with his forefinger; after several attempts he succeeded in picking up a pin from the table. He had never suffered from pains in the stomach or limbs; he was giddy at times, but not more so in the dark. He walked fairly well, without any ataxy of movement. On examination of the chest, there was dulness over the upper lobe of the right lung in front as far as the fourth rib, with, over the dull area, tenderness, harshness of breath-sounds, and increase of vocal resonance. Heart was normal; pulse 96, regular; the abdominal organs were normal; the tongue was furred and tremulous.

In the legs no tremor was observed, though he said that they felt tremulous when he walked. There was slight but distinct rigidity of leg-muscles. The knee-jerks, plantar, cremasteric, and superficial abdominal reflexes were exaggerated; there was slight ankle-clonus on the right side, but not on the left; and the front-tap contraction was readily obtained. In the arms the bicipital and tricipital tendon reflexes were exaggerated, and the muscles responded too readily to a smart tap on the lower ends of the radius or ulna. There was no wasting of the muscles, and the electrical reactions were normal.

There was no loss of sensation, but there was an area of hyperæsthesia extending round the right side over the 7th and 8th intercostal spaces, and also over the epigastrium. There was tenderness to pressure over the first three dorsal spines. As to special senses, he complained of a bad smell; but smell and taste seemed to be perfectly normal, so that this must have been subjective. His sight was good: the pupils equal, acting both to accommodation and to light; the fundus was quite healthy. There was nystagmus, which was nearly constant; generally the movements were in the vertical, but occasionally in the horizontal, direction, more especially when the eyes were directed laterally. There was no oculomotor paralysis. He was slightly deaf. He complained of passing a large quantity of water and of frequent micturition; but it was found that he passed about 60 ozs. in the 24 hours, of normal composition. The speech was unaffected, and did not present the scanning character so common in this disease.

## Figures and Tables

**Figure f1:**
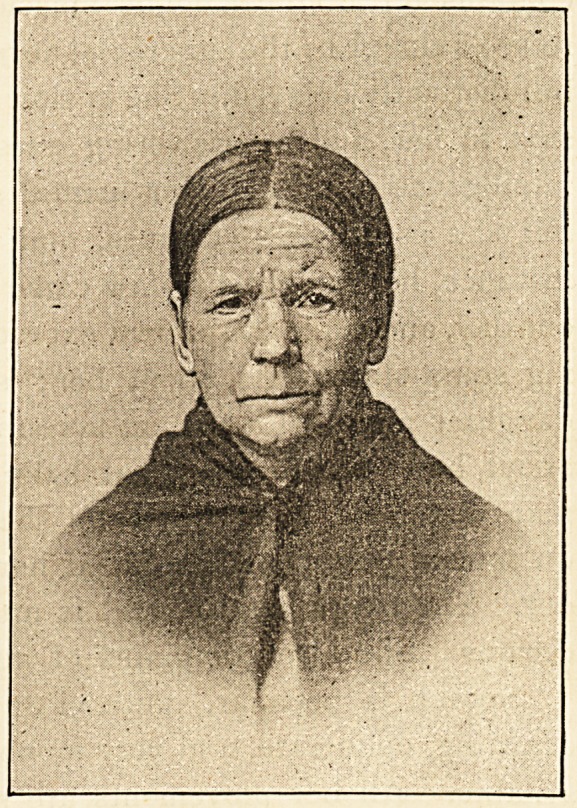


**Figure f2:**
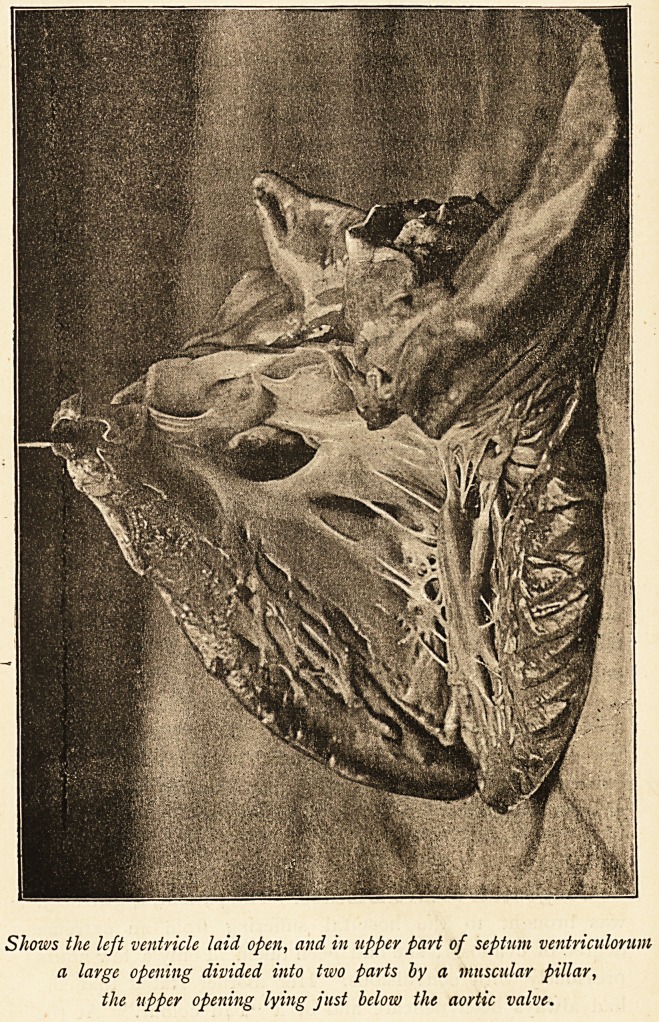


**Figure f3:**